# Differences of cardiac output measurements by open-circuit acetylene uptake in pulmonary arterial hypertension and chronic thromboembolic pulmonary hypertension: a cohort study

**DOI:** 10.1186/1465-9921-13-18

**Published:** 2012-03-12

**Authors:** Martin Schwaiblmair, Christian Faul, Wolfgang von Scheidt, Thomas M Berghaus

**Affiliations:** 1 1. Department of Internal Medicine, Klinikum Augsburg, Ludwig-Maximilians-University Munich, Stenglinstrasse 2, D-86156 Augsburg, Germany

**Keywords:** Pulmonary arterial hypertension, Chronic thromboembolic pulmonary hypertension, Cardiac output, Single-breath open-circuit acetylene uptake, Thermodilution

## Abstract

**Background:**

As differences in gas exchange between pulmonary arterial hypertension (PAH) and chronic thromboembolic pulmonary hypertension (CTEPH) have been demonstrated, we asked if cardiac output measurements determined by acetylene (C_2_H_2_) uptake significantly differed in these diseases when compared to the thermodilution technique.

**Method:**

Single-breath open-circuit C_2_H_2 _uptake, thermodilution, and cardiopulmonary exercise testing were performed in 72 PAH and 32 CTEPH patients.

**Results:**

In PAH patients the results for cardiac output obtained by the two methods showed an acceptable agreement with a mean difference of -0.16 L/min (95% CI -2.64 to 2.32 L/min). In contrast, the agreement was poorer in the CTEPH group with the difference being -0.56 L/min (95% CI -4.96 to 3.84 L/min). Functional dead space ventilation (44.5 ± 1.6 vs. 32.2 ± 1.4%, p < 0.001) and the mean arterial to end-tidal CO_2 _gradient (9.9 ± 0.8 vs. 4.1 ± 0.5 mmHg, p < 0.001) were significantly elevated among CTEPH patients.

**Conclusion:**

Cardiac output evaluation by the C_2_H_2 _technique should be interpreted with caution in CTEPH, as ventilation to perfusion mismatching might be more relevant than in PAH.

## Introduction

The assessment of cardiac output is a crucial factor in the risk stratification and management of patients with pulmonary hypertension (PH) as it is directly related to the clinical severity of the disease as well as being one of the most important prognostic factors [1].

Several methods have been introduced to measure cardiac output in humans. The thermodilution technique has been validated against the direct *Fick *method, which represents the "gold standard" when evaluating cardiac output [2]. It is used routinely to assess cardiac output in PH patients. However, the thermodilution method is an invasive technique requiring right heart catheterization of the patient. A reliable non-invasive method to determine the cardiac output would allow serial measurements and, thus, would facilitate the follow-up management of PH patients. Among the non-invasive techniques the acetylene (C_2_H_2_) rebreathing method has been validated against different other techniques and has gained wide acceptance [2-4]. A drawback to the method, however, is the build-up of carbon dioxide (CO_2_) as a result of rebreathing. Therefore, open-circuit methods have been developed [5,6]. C_2_H_2 _is a non-toxic, inert gas that has a low solubility in lung tissue but a high solubility in blood. When inhaled, C_2_H_2 _is rapidly taken up in the pulmonary blood stream at a rate proportional to the effective pulmonary blood flow. The rate of disappearance allows calculating the cardiac output in the absence of a relevant pulmonary shunt blood flow. Yet, this technique is expected to fail whenever there are conditions that may affect the distribution of the gas in the lungs. The alveolar distribution of C_2_H_2 _should not be affected in PH patients. However, significant differences in gas exchange between pulmonary arterial hypertension (PAH) and chronic thromboembolic pulmonary hypertension (CTEPH) have been demonstrated recently, most likely resulting from different lung perfusion patterns and additional thromboembolic vascular occlusion in CTEPH [7].

We therefore aimed to clarify if cardiac output measurements done by a single-breath open-circuit C_2_H_2 _uptake method significantly differ in PAH and CTEPH patients when compared to the cardiac output assessment performed by thermodilution.

## Materials and Methods

### Study design and study population

This retrospective cohort study included patients who were referred to our PH centre between January 2007 and December 2010. All study participants fulfilled the diagnostic criteria for PAH or CTEPH according to current guidelines [1]. All patients underwent right heart catheterization to establish the diagnosis. CTEPH was ruled out or confirmed by ventilation/perfusion scan or pulmonary angiography in every patient. No subject in the study received any specific pulmonary artery medication upon inclusion in the trial. Most of our study participants were referred to our hospital for the evaluation of PH or unexplained dyspnea on exertion. Therefore, all patients were extensively diagnosed for the presence of lung disease. Patients with pulmonary comorbidity (such as asthma, chronic obstructive pulmonary disease, lung fibrosis or other symptomatic interstitial lung diseases) or concomitant extra-cardiac diseases limiting exercise performance were excluded. The catheter examination was part of the routine diagnostic work-up in all patients. All procedures adhered to commonly accepted ethical guidelines and written informed consent was obtained from every patient.

### Lung Function Tests

Pulmonary function tests included spirometry, body plethysmography and measurement of diffusing capacity employing the single-breath method (Master Screen Body and MS-PFT, Jaeger, Cardinal Health, USA). Each parameter was calculated as percentage of the predicted one. The following parameters were measured: forced vital capacity (FVC), total lung capacity (TLC), forced expiratory volume in one second (FEV1) and diffusing capacity for carbon monoxide (TLco). Blood gas analysis (ABL 725, Radiometer, Copenhagen, Denmark) was performed in arterialized capillary blood from the ear lobe without supplemental oxygen (O_2_).

### Cardiopulmonary exercise testing

Cardiopulmonary exercise testing (CPET) was performed using a standardized protocol [6]. Work rate was continuously increased by 5 - 15 watts/min to a maximum tolerated level on an electromagnetically braked cycle ergometer (ViaSprint 150 p, Ergoline, Germany). Patients were encouraged to exercise until symptoms were intolerable. Blood gas analysis was done at rest and during peak exercise. Heart rate was monitored continuously and non invasive blood pressure was taken every 2 minutes. The maximum work rate was recorded. O_2 _uptake (VO2), minute ventilation (Ve) and CO_2 _output (VCO2) were measured breath by breath using an adult facemask (Vmax spectra 229 D, Sensor Medics, USA). O_2 _pulse, alveolar-arterial O_2 _difference (AaDO2) and functional dead space ventilation (Vd/Vt) were calculated as described by *Wasserman *et al. [8]. The anaerobic threshold (AT) was chosen at the peak VO2 at which the ventilatory equivalent for O_2 _(Ve/VO2) increased while the ventilatory equivalent for CO_2 _(Ve/VCO2) decreased or remained constant. Peak VO2 was defined as the value of averaged data during the final 15 seconds of exercise. The Ve/VCO2 slope was determined as the linear regression slope of Ve and VCO2 from the start of exercise until the respiratory compensation point (the time point at which ventilation is stimulated by acidaemia and the end-tidal CO_2 _(etCO2) begins to decrease).

### Right heart catheterization and thermodilution

Patients received no medication on the morning of the procedure, resulting in a discontinuation of treatment of at least 12 hours. A thermodilution catheter (7.5 F quadruple-lumen, balloon-tipped, flow-directed, "S" Tip Swan-Ganz Catheter, Edwards Lifesciences, Irvine, USA) was inserted via the right or left femoral vein. Hemodynamic measurements were performed in supine position and included heart rate, pressure in wedge-position (PCWP), pulmonary arterial pressure (PAP) and right atrium pressure (RAP). O_2 _saturation (SO2) was measured in mixed venous blood samples (ABL 725, Radiometer, Copenhagen, Denmark). The cardiac output was measured by thermodilution with 10 ml of sterile, ice-cold isotonic (0.9%) saline, which was injected through the right atrial lumen of the catheter; the drop in temperature at the distal thermistor was then recorded. The injectate temperature was determined by a thermistor which was placed directly behind the right atrial inlet of the catheter. Cardiac output was calculated using a computer system (Com-2, Cardiac Output Computer, Edwards Lifesciences, Irvine, USA). In each patient, a minimum of 3 measurements were performed; the mean value was calculated if the variability of values was less than 10%. The pulmonary vascular resistance (PVR) was calculated using a standard formula [PVR = (mean PAP - PCWP)/cardiac output].

### C_2_H_2 _Technique

C_2_H_2 _uptake was measured by an open-circuit single-breath, slow-expiration method using a commercially available system (Vmax spectra 229 D, Sensor Medics, USA). The precision and the reliability of this system have been validated by others [5] and the instructions of the manufacturer were followed in detail. Briefly, the patients were instructed to breathe through the mouthpiece of the apparatus. The nostrils were occluded with a nose clip. The inhaled gas was a mixture of 0.298% methane, 0.300% carbon monoxide, 0.300% C_2_H_2_, 21.100% O_2 _and 78.002% nitrogen. The manoeuvre started at end-expiration with a maximal inspiration. The breath hold time was 1-3 seconds. Then the patients slowly exhaled the total volume. Thereafter, cardiac output was calculated by an integrated computer from the disappearance curve of C_2_H_2_. In each patient, a minimum of 3 measurements were performed; the mean value was calculated if the variability of values was less than 10%. The procedure was tolerated without any problems by all patients.

### Statistical Analysis

Statistical analysis was performed with SPSS software for Windows version 12.0 (SPSS, IBM Inc., Chicago, USA). All data are presented as mean ± standard error of mean (SEM). The results obtained from the PAH and CTEPH group were compared using the Student's *t*-test for unpaired probes. The agreement between the thermodilution and C_2_H_2 _method was analyzed as described by *Bland *and *Altman *[9]. Agreement bias was expressed as the mean of the differences obtained by the different techniques. The limits of agreement were expressed as the mean ± 2 SD, and the 95% confidence interval (95% CI) of the bias was calculated. Simple linear regression analysis was performed in order to compare the quality of agreement between the cardiac output measurements obtained by the different techniques in both cohorts. A probability value of less than 0.05 was considered statistically significant; all reported p values are two-tailed.

## Results

### Clinical Characteristics of the study population

A total of 104 patients were included in the study (Table 1). 72 patients were diagnosed having PAH and 32 study participants were suffering from CTEPH. CTEPH patients were significantly older than subjects in the PAH subgroup (70.4 ± 1.4 vs. 52.5 ± 2.3 years, p < 0.001). Gender and body mass indices (BMI) were equally distributed in both groups.

**Table 1:** Clinical characteristics, lung function testing, and hemodynamics

	All n = 104	PAH n = 72	CETPH n = 32	p
**Characteristics**				
Age (years)	58.1 ± 1.8	52.5 ± 2.3	70.4 ± 1.4	< 0.001
Female/male (n)	61/43	47/25	20/12	0.898
BMI (kg/m^2^)	25.9 ± 0.5	25.4 ± 0.6	27.4 ± 0.8	0.192
**Lung function**				
FVC (L)	2.98 ± 0.08	3.07 ± 0.10	2.73 ± 0.16	0.285
FVC (%)	89.9 ± 1.6	90.4 ± 1.8	86.9 ± 2.6	0.641
TLC (L)	5.41 ± 0.11	5.37 ± 0.14	5.47 ± 0.19	0.209
TLC (%)	98.1 ± 1.5	98.0 ± 1.8	95.8 ± 2.7	0.880
FEV1 (L/s)	2.09 ± 0.07	2.21 ± 0.08	1.80 ± 0.11	0.005
FEV1/FVC (%)	70.2 ± 1.0	72.2 ± 1.0	65.4 ± 2.1	0.002
TLco (%)	61.0 ± 2.1	59.9 ± 2.7	63.8 ± 2.9	0.943
pO_2 _at rest (mmHg)	61.2 ± 1.1	63.8 ± 1.4	54.6 ± 1.5	< 0.001
pCO_2 _at rest (mmHg)	30.6 ± 0.5	29.8 ± 0.6	32.3 ± 0.7	0.015
**Hemodynamics**				
mPAP (mmHg)	47.0 ± 1.4	46.0 ± 1.7	51.1 ± 2.2	0.255
Cardiac output (L/min)	3.96 ± 0.15	4.09 ± 0.14	3.94 ± 0.18	0.001
CI (L/min/m^2^)	2.27 ± 0.06	2.38 ± 0.07	2.02 ± 0.09	0.004
PVR (dyne •s/cm^5^)	791 ± 31	779 ± 37	843 ± 58	0.181
mRAP (mmHg)	7.2 ± 0.5	6.1 ± 0.5	10.0 ± 1.3	0.001
PCWP (mmHg)	8.8 ± 0.3	8.2 ± 0.4	10.6 ± 0.7	0.002
SVO_2 _(%)	58.3 ± 0.9	58.9 ± 0.9	56.7 ± 1.9	0.527

Data are shown as means ± SEM.

### Lung Function Testing

Lung function tests showed no relevant restrictive lung disease in both groups (Table 1). The FEV1 and the FEV1/FVC ratios were located on the lower limit of normal (2.09 ± 0.07 L/s and 70.2 ± 1.0%, respectively) with significant lower values in the CTEPH group (1.80 ± 0.11 vs. 2.21 ± 0.08 L/s, p = 0.005 and 65.4 ± 2.1 vs. 72.2 ± 1.0%, p = 0.002, respectively).

### Hemodynamics

Mean PAP was elevated with 47.0 ± 1.4 mmHg with a normal PCWP of 8.8 ± 0.3 mmHg in both groups (Table 1). However, the cardiac output and cardiac indices (CI) were significantly lower in the CTEPH group (3.68 ± 0.18 vs. 4.09 ± 0.14 L/min, p = 0.001 and 2.02 ± 0.09 vs. 2.38 ± 0.07 L/min/m^2^, p = 0.004, respectively).

### CPET

Both groups showed a reduction in work capacity of 51.0 ± 2.5% with a diminished VO2 of 62.9 ± 2.3% (16.0 ± 0.6 ml/min/kg), a reduced O2 pulse of 9.3 ± 0.3 ml/min/beat and an elevated Ve/VO2 of 41.2 ± 1.0 and Ve/VCO2 of 48.3 ± 1.0 at the AT of 10.4 ± 0.4 ml/min/kg (Table 2). In addition, we observed an increased AaDO2 of 50.7 ± 1.3 mmHg and an elevated Vd/Vt of 35.8 ± 1.2% during peak exercise with an arterial to end-tidal CO_2 _gradient (a-etCO2) of 5.7 ± 0.5 mmHg. Ve/VCO2 slope amounted to 49.6 ± 1.6 with a PetCO2 of 23.9 ± 0.6 mmHg at peak exercise. In comparison to the PAH group, the Ve/VCO2 was found to be significant higher in the CTEPH group (51.7 ± 1.4 vs. 47.1 ± 1.3, p = 0.036). In addition, the Vd/Vt and the a-etCO2 were also significantly elevated among CTEPH patients compared to the PAH subgroup (44.5 ± 1.6 vs. 32.2 ± 1.4%, p < 0.001 and 9.9 ± 0.8 vs. 4.1 ± 0.5 mmHg, p < 0.001, respectively).

**Table 2:** Cardiopulmonary exercise testing

	All n = 104	PAH n = 72	CETPH n = 32	p
W (watts)	57.7 ± 3.4	63.4 ± 4.4	44.0 ± 4.6	0.011
W (%)	51.0 ± 2.5	52.8 ± 2.9	43.8 ± 4.6	0.192
VO2 (ml/min)	1111 ± 43	1150 ± 55	1033 ± 69	0.513
VO2 (%)	62.9 ± 2.3	61.2 ± 2.9	66.1 ± 3.9	0.638
VO2 (ml/min/kg)	16.0 ± 0.6	16.7 ± 0.8	14.0 ± 0.9	0.044
AT (ml/min/kg)	10.4 ± 0.4	11.0 ± 0.5	9.1 ± 0.6	0.019
O2 pulse (ml/min/beat)	9.3 ± 0.3	8.9 ± 0.3	10.1 ± 0.6	0.137
Ve (L/min)	61.4 ± 2.0	63.3 ± 2.6	63.3 ± 2.6	0.416
Ve/VO2	41.2 ± 1.0	40.8 ± 1.3	42.3 ± 1.4	0.299
Ve/VCO2	48.3 ± 1.0	47.1 ± 1.3	51.7 ± 1.4	0.036
AaDO2 (mmHg)	50.7 ± 1.3	50.4 ± 1.7	51.5 ± 1.6	0.775
Vd/Vt (%)	35.8 ± 1.2	32.2 ± 1.4	44.5 ± 1.6	< 0.001
a-et CO2 (mmHg)	5.74 ± 0.5	4.1 ± 0.5	9.9 ± 0.8	< 0.001
Ve/VCO2 slope	49.6 ± 1.6	48.6 ± 2.0	53.2 ± 2.3	0.145
RER	1.08 ± 0.01	1.09 ± 0.01	1.05 ± 0.02	0.143

Data are shown as means ± SEM.

### Comparison of cardiac output measurements by the C_2_H_2 _method and thermodilution

**Figure 1:**
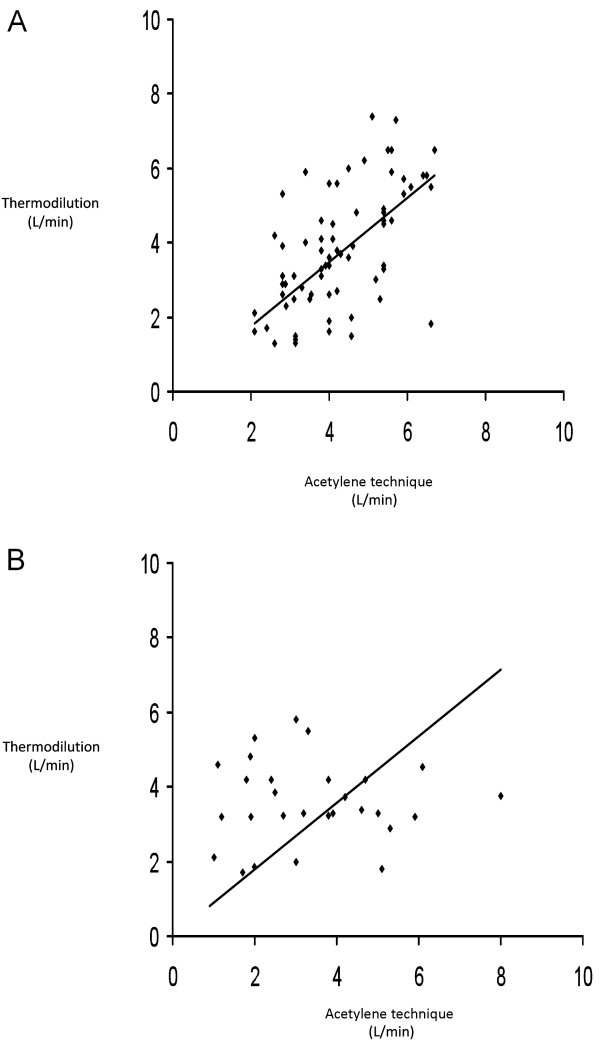
**Cardiac output measurements by thermodilution compared with results obtained by the acetylene technique in patients with pulmonary arterial hypertension (a) and chronic thromboembolic hypertension (b)**. The *solid line *represents the bisecting line.

**Figure 2:**
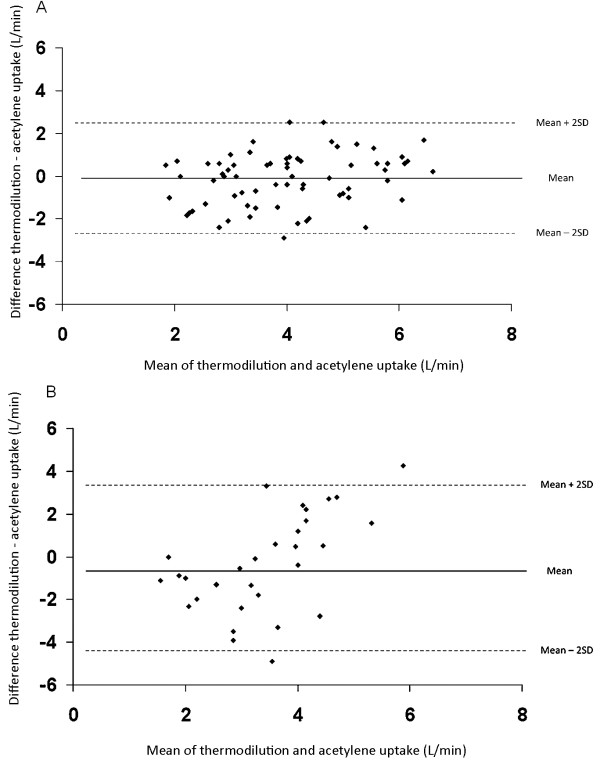
**Individual differences in cardiac output between thermodilution and acetylene uptake in patients with pulmonary arterial hypertension (a) and chronic thromboembolic hypertension (b), plotted against the average corresponding values (L/min)**. The *solid line *represents the mean or bias of the differences; the *dashed lines *represent the upper and normal limits of agreement.

The average cardiac output as determined by thermodilution was 3.96 ± 0.15 L/min and 3.68 ± 0.22 L/min by the C_2_H_2 _technique for the whole study population. Among PAH patients, the mean cardiac output was 4.09 ± 0.14 L/min measured by thermodilution and 3.94 ± 0.18 L/min evaluated by C_2_H_2 _uptake. In the CTEPH group, mean cardiac output was determined to be 3.68 ± 0.18 L/min by thermodilution and 3.10 ± 0.32 L/min by the C_2_H_2 _technique. Figure 1 plots the results obtained by thermodilution with those obtained by C_2_H_2 _uptake. Figure 2 shows the *Bland & Altman *plot of the differences between thermodilution and C_2_H_2 _technique against the mean of both values. In PAH patients the results obtained by the two methods showed an acceptable agreement with the mean difference being -0.16 L/min (95% CI -2.64 to 2.32 L/min). In contrast, the agreement was poorer in the CTEPH group with the difference being -0.56 L/min (95% CI -4.96 to 3.84 L/min). Simple linear regression analysis revealed that values for cardiac output measured by thermodilution and C_2_H_2 _uptake were significantly associated in the PAH subgroup (R^2 ^= 0.122, p = 0.004). In contrast, cardiac output measurements obtained by the different techniques in the CTEPH cohort were not significantly associated (R^2 ^= 0.032, p = 0.35).

## Discussion

Our study revealed an acceptable agreement of the cardiac output assessment determined by thermodilution and C_2_H_2 _uptake in PAH patients, although we observed a large splay of single measurements. These differences, however, were not larger than those reported from patients with other cardiopulmonary diseases [10]. Our findings are in accordance with an earlier trial by *Hoeper *et al. who reported that thermodilution and C_2_H_2 _rebreathing are equally accurate in patients with idiopathic PAH when being compared with the direct Fick method [2]. In contrast, our study demonstrated that the agreement between thermodilution and the C_2_H_2 _technique was poor in patients suffering from CTEPH.

Several issues need to be discussed in this context. In our study CTEPH patients had lower CI values compared to the PAH subgroup. As C_2_H_2 _uptake tends to underestimate cardiac output in our CTEPH cohort and thermodilution might overestimate cardiac output in the presence of a low CI [11], the poorer agreement of both methods could be explained by a wider divergence of measurements. However, another trial has challenged this hypothesis indicating that thermodilution is equally accurate in a broad spectrum of cardiac output values [2].

Furthermore, mean FEV1 and the averaged FEV1/FVC ratios were significantly reduced in the CETPH cohort, possibly explaining the poorer agreement of both methods. As patients with concurrent lung diseases were excluded from our trial and both groups had similar mean BMI values, these findings might be explained by the older mean age in the CETPH subgroup. As the alveolar distribution of C_2_H_2 _might be affected by the reduction of FEV1/FVC, cardiac output measurements obtained by the C_2_H_2 _uptake method are probably less accurate in the CTEPH cohort.

In addition to that, an impaired gas exchange might contribute to the poorer agreement of both techniques in the CTEPH cohort, as the C_2_H_2 _technique is expected to fail in the presence of a relevant mismatching of ventilation to perfusion. Indeed, we found evidence of an uneven distribution of ventilation to perfusion in CTEPH patients primarily due to two different mechanisms, increased dead space ventilation and heterogeneous pulmonary perfusion.

Although there are some reports indicating that ventilation/perfusion matching is relatively well preserved in both conditions [12-14], there is evidence that increased ventilation/perfusion ratios in CTEPH are primarily caused by augmented dead space fractions [15,16]. In our study Vd/Vt at peak exercise was significantly increased in CTEPH compared to PAH. These findings are in accordance with the report of *Zhai *and colleagues, who were able to show that significant differences in gas exchange exist between CTEPH and PAH due to differences in Vd/Vt [7]. Dead space ventilation in pulmonary thromboembolism increases the gradient between arterial and end-tidal CO_2 _[17]. Correspondingly, we found the mean a-etCO2 gradient to be significantly elevated among CTEPH patients compared to the PAH subgroup in our study. Similar findings have recently been made by *Scheidl *and co-workers [18], who found increased capillary to end-tidal CO_2 _differences in patients with CTEPH compared to those suffering from idiopathic PAH. Increased dead space ventilation might be the result of a more uneven perfusion pattern in CTEPH patients, possibly due to additional thrombus formation and a more proximal vascular occlusion, leading to heterogeneous perfusion defects [19]. Areas with diminished blood flow and areas with increased blood flow coexist, while ventilation is more or less homogeneously distributed. As a result, there are areas with an increased ventilation/perfusion ratio or even dead space ventilation and others with a low ventilation/perfusion ratio. In contrast, PAH is characterized by an obstructive vasculopathy that bilaterally involves distal, medium to small size muscular arteries [20], possibly resulting in a more balanced distribution of perfusion and ventilation. Therefore, we speculate that compared to PAH patients, increased heterogeneity in pulmonary blood flow in comparison to ventilation might contribute to an inaccurate determination of cardiac output by the C_2_H_2 _method in CTEPH.

Our study is limited by the fact that cardiac output measurements determined by thermodilution and the C_2_H_2 _method were not performed simultaneously. Thus, the splay of single measurements might partially be explained by the physiological variations of cardiac output during the day. However, both techniques were performed shortly after each other without any change of medication in all study participants in order to minimize substantial fluctuations. Moreover, the results might have been influenced by different body positions, as right heart catheterizations were performed in supine position while patients were sitting when the C_2_H_2 _technique was performed. Thus, measurements by the C_2_H_2 _uptake method might tend to underestimate cardiac output, as preload decreases when subjects sit up. This circumstance possibly explains some of the very low cardiac output values measured by C_2_H_2 _uptake. Finally, we didn't evaluate the degree of tricuspid regurgitation, which is commonly present in patients with PH and might have influenced the accuracy of thermodilution in our study participants [21]. As a consequence, thermodilution might have underestimated the cardiac output in the presence of severe tricuspid regurgitation.

## Conclusions

Despite these limitations we conclude the following: in contrast to PAH, the agreement of cardiac output measurements determined by the thermodilution and the C_2_H_2 _uptake method is poorer in CTEPH. The evaluation of cardiac output by the C_2_H_2 _technique should be interpreted with caution in CTEPH patients, as ventilation to perfusion mismatching might be more relevant in CTEPH than in PAH.

## Abbreviations

BMI: body mass index; FVC: forced vital capacity; TLC: total lung capacity; FEV1: forced expiratory volume in one second; TLco: diffusing capacity; pO_2_: oxygen partial pressure; pCO_2_: carbon dioxide partial pressure; mPAP: mean pulmonary artery pressure; CI: cardiac index; PVR: pulmonary vascular resistance; mRAP: mean right atrial pressure; PCWP: pulmonary capillary wedge pressure; SVO2: mixed venous O_2 _saturation; W: work capacity; VO_2_: peak O_2 _uptake; AT: anaerobic threshold; Ve: peak minute ventilation; Ve/VO2: O_2 _equivalent at anaerobic threshold; Ve/VCO2: CO_2 _equivalent at anaerobic threshold; AaDO2: alveolar-arterial O_2 _difference at peak exercise; Vd/Vt: functional dead space ventilation at peak exercise; a-et CO2: arterial to end-tidal CO_2 _gradient at peak exercise; Ve/VCo2 slope: slope of minute ventilation versus CO_2 _output; RER: respiratory exchange ratio at peak exercise.

## Authors' contributions

Drs. Schwaiblmair, von Scheidt and Berghaus conceived and designed the study. Drs. Schwaiblmair, Faul and Berghaus acquired the study data. Dr. Schwaiblmair performed the statistical analysis. Drs. Schwaiblmair and Berghaus drafted the article. All authors participated in interpreting the data and revised the manuscript for important intellectual content. All authors approved the final version of the manuscript.

## Disclosure

The study was supported by a grant of *Pfizer*. The supporting source had no involvement in the study design, data collection, analysis or interpretation of data, in the writing of the report, and in the decision to submit the manuscript for publication.
